# Pb Transfer Preference of Arbuscular Mycorrhizal Fungus *Rhizophagus irregularis* in *Morus alba* under Different Light Intensities

**DOI:** 10.3390/jof8111224

**Published:** 2022-11-20

**Authors:** Wei Ren, Haoqiang Zhang, Xiaoxia Jin, Hongchao Huang, Linxi Zhou, Tingying Xu, Ming Tang

**Affiliations:** 1 College of Forestry, Northwest A&F University, Yangling 712100, China; 2 Boone Pickens School of Geology, Oklahoma State University, Stillwater, OK 74078, USA; 3 State Key Laboratory of Conservation and Utilization of Subtropical Agro-Bioresources, Guangdong Laboratory for Lingnan Modern Agriculture, College of Forestry and Landscape Architecture, South China Agricultural University, Guangzhou 510642, China

**Keywords:** lead translocation, mycorrhizal symbiosis, mycorrhizal hyphae networks, resource allocation, shading

## Abstract

Arbuscular mycorrhizal (AM) fungi can improve the lead (Pb) tolerance of host plants and accumulate intensive Pb in mycorrhizal roots. However, the detailed contribution of AM fungal extraradical hyphae to the plants’ Pb uptake remains unknown. In this study, mulberry (*Morus alba*) colonized by the AM fungus (*Rhizophagus irregularis*) with light treatments were linked by fungal extraradical hyphae using a three-compartment system (pot test), and their differences in responding to Pb application were compared. Shading inhibited mulberry photosynthesis and the growth of mulberry. In this study, Pb application did not affect the colonization of *R. irregularis* when symbiosis had already formed as the root was not exposed to Pb during the colonization and formation of the AM fungal hyphae network. The *R. irregularis* preferred to transfer more Pb to the unshaded mulberry than to the shaded mulberry, a condition capable of providing more C supply for fungal survival than to low-light mulberry. The Pb transferred through the mycorrhizal pathway to mulberry had low mobility and might be compartmented in the root by *R. irregularis* until exceeding a threshold. The relatively high expressions of MaABCG16 with high Pb concentrations in plants suggest that MaABCG16 might play an important role in Pb translocation.

## 1. Introduction

The heavy metal contamination of soils resulting from urban development and industrial processes is increasingly becoming a serious environmental problem around the world [1,2]. As a non-essential element, lead (Pb) accumulated in soils has become an immense risk for animals and humans due to its toxicity even at low concentrations [3,4,5]. Short-term exposure to Pb can cause brain and kidney damage, while long-term exposure can harm the central nervous and reproductive system, which can even lead to death [6,7,8]. China has the second largest Pb reserves in the world, producing an estimated two million metric tons of Pb in 2021 [9]. Soil Pb contamination is becoming one of the major environmental problems to be solved urgently in China [10,11]. Mycorrhizoremediation, an emerging bioremediation strategy that uses mycorrhizal fungi and adapted plant species, can be an efficient and non-invasive way to remediate heavy metal pollutants from the environment [12,13,14,15].

Arbuscular mycorrhizal (AM) fungi, widespread in the rhizosphere of contaminated soils, can improve the contaminant tolerance and growth of host plants in these environments [16,17,18]. The AM fungi can improve the uptake of mineral nutrients of their host plants via increasing the absorption surface area and mobilizing less soluble mineral nutrients such as phosphorus (P), potassium (K), nitrogen (N), and some micronutrients [19]. In return, the host plant can provide the necessary carbon (C) for AM fungi to grow [20,21,22]. Plant C investment in this mutualistic symbiosis stimulates the enhancement of the photosynthetic rates, transpiration flow, and water uptake of mycorrhizal plants [23,24,25]. Under Pb stress, the increment of plant growth can dilute the contaminant concentration within the plant, which leads to an improved Pb tolerance of the host plants and a decrease in the Pb concentrations in soils [26,27]. In addition, AM fungi can reduce Pb toxicity to their host plant by modulating the plant antioxidant enzyme system to protect the host plant [26,28,29]. *Rhizophagus irregularis* can form mycorrhizae with many terrestrial plants [29,30] including *Morus alba*, according to our previous unpublished research. In addition, *R. irregularis* has been reported to be capable of improving the heavy metal tolerance of host plants. Thus, *R. irregularis* can serve as a model strain to reveal the detailed interactions between AM fungi and *M. alba* under Pb stress [30,31].

Mulberry (*M. alba*) is a fast-growing and perennial deciduous woody tree that can adapt to various types of environments [32,33]. Prince et al. [34] and Zhou et al. [35] assessed the bio-mobility of heavy metals (i.e., copper (Cu), cadmium (Cd), and Pb) in a soil–plant–insect system using the mulberry–silkworm food chain and observed that heavy metals were accumulated by mulberry roots, with a limited amount transported to the leaves and larvae. Coupled with its large biomass and deep root, mulberry shows high potential in the extraction or detoxification of heavy metals from polluted soils [36,37]. It has been observed that the promoting effect of mycorrhizal fungi on mulberry also exists under Cd stress and AM mulberry copes better with Cd stress than non-inoculated mulberry [38]. To date, the application and mechanism of AM fungal mulberry in Pb remediation have not been resolved systematically.

Several studies have demonstrated that the transpiration of host plants is the driving force for the transport of nutrients (e.g., P, K, and N) from AM fungi to host plant roots [39,40,41] and the suppression of host plant transpiration can decelerate nutrient translocation in proportion to the level of suppression [42]. A previous study has proposed that AM fungi are capable of transferring Pb to the host plant by stimulating transpiration flow [27]. However, a detailed understanding of how Pb is transferred to mycorrhizal roots remains unknown. Do AM fungi have a preference for Pb translocation in plants with different transpiration? Does the transfer of Pb by AM fungi to the host plant follow the biological market theory, in which the AM fungi prefer to transfer nutrients to the host plant with high C payback [20]? Thus, a systematic investigation of Pb transport in AM fungi and its role in improving host plant metal tolerance is warranted to answer these questions.

ATP-binding cassette (ABC) proteins are powerful transporters to drive the exchange of compounds across many different biological membranes using the energy released from ATP hydrolysis [43,44]. Fan et al. [45] found that the expression level of *AtPSE1*, a member of ABCs, is related to plant Pb tolerance. Bhuiyan et al. [46] also found that the overexpression of the ABCs of *Arabidopsis thaliana* in *Brassica juncea* conferred enhanced tolerance and the accumulation of heavy metals in the plants. Gonzalez-Guerrero et al. [47] cloned ABC genes of AM fungus from the extraradical hyphae of the AM fungus and found that it was upregulated by heavy metals. These studies suggest that the important roles of ABCs in Pb transfer deserve further study. Moreover, the element exchange between AM and the host plant requires the participation of aquaporins (AQPs), the knockdown of AQP in AM fungi as well as the suppression of host transpiration decelerated element translocation in proportion to the levels of knockdown and suppression [42]. Therefore, the involvement of fungal AQPs and ABCs in Pb transfer should be examined.

Light is necessary for plant photosynthesis. When the light received by plants is inhibited, the photosynthetic efficiency of plants decreases. Several studies have shown that plant investment in mycorrhizal fungi decreased as light decreased [48,49]. It remains unknown whether the AM fungal transfer of non-essential elements such as Pb would be affected when C investment in AM fungi is reduced. To verify the contribution of AM fungal extraradical hyphae to plant Pb uptake and assess the Pb transfer preference of AM fungi to plants with different light intensities, a three-compartment system was established in this study to link two mulberry seedlings (under the same or different light intensities) with AM fungal extraradical hyphae. The three-compartment system creates a separate compartment for the fungal hyphae where only the hyphae are exposed to the heavy metals and any heavy metals received by the plant should be transferred through the hyphae. The research on *Cucumis sativus*, *Phaseolus vulgaris*, and *Zea mays* have described the contribution of AM fungus (*Glomus mosseae*) on Cd uptake using a similar three-compartment system and proved that three-compartment is an ideal system to separate and understand the contribution of AM fungal extraradical hyphae [50,51]. We hypothesized that: (1) the extraradical hyphae of AM fungi prefer to transfer Pb (absorbed from soil) to the host plant with strong photosynthesis when only AM fungal extraradical hyphae are exposed to Pb; and (2) the limitation of light intensity can inhibit the transfer of Pb between AM fungi and mulberry. To our knowledge, this is the first study using a three-compartment system to verify the Pb transfer capacity of AM fungal extraradical hyphae, which is of great importance in the understanding of heavy metal interaction within the AM fungi–host plant system.

## 2. Materials and Methods

### 2.1. Plant Material, Growth Substrates, and AM Fungal Inoculum

Seeds of mulberry (*M. alba* var. Guiyou 12) were provided by the General Extension Station of Sericulture Technology of Guangxi. The seeds were sterilized with 75% alcohol for 30 s and 1% (*v*/*v*) sodium hypochlorite for 8 min. After being washed three times with sterile distilled water, the seeds were put on sterilized wet filter paper for 7 days to germinate. Then, the seedlings were transplanted into plastic seeding pots with the sterilized substrate (sand: vermiculite = 1:1, *v*:*v*) in a greenhouse with 28 °C/24 °C day/night temperatures under 16 h daylight and 40–60% humidity.

After two weeks, uniformed seedlings were planted on the left and the right of a three-compartment system in which both the left and right compartments were inoculated with AM inoculum (*R. irregularis*). The three-compartment system was made of acrylic plates and was stuck with an acrylonitrile-butadiene-styrene (ABS) plastic adhesive (Supplementary Figure S1A). The compartments were separated by a nylon net (38 μm) to retain the plant roots but allow the AM fungal hyphae to pass. The left and right compartments were filled with 300 g growth substrate with 10 g AM inoculum. The middle compartment (i.e., hyphal compartment) was filled with isopycnic sand (440 g) to allow the hyphae to extend. This three-compartment system formed a hyphae bridge between the plants in the left and right compartments, which created an independent hyphal compartment for Pb application without directly exposing the plants to Pb. This experimental setup was specifically designed to evaluate the preference of the AM fungi for Pb transfer to plants.

The growth substrate was a mixture of sand and vermiculite (1:1; *v*:*v*) with the natural contents of available nitrogen (10.78 mg·kg^−1^), available phosphorus (2.49 mg·kg^−1^), and available potassium (24 mg·kg^−1^). The sand was sieved through a 2 mm sieve, thoroughly washed with tap water, and then sterilized at 170 °C for 4 h. The vermiculite was autoclaved at 121 °C (0.11 MPa) for 2 h for sterilization.

The AM inoculum of *R. irregularis* (Bank of Glomales in China, No. BGC BJ09), which consisted of a sandy substrate that contained spores (approximately 52 spores per gram), mycelium, and colonized root fragments, was provided by the Beijing Academy of Agriculture and Forestry Sciences (Beijing, China) and enriched in pot cultures of *Zea mays* Linn. The AM inoculum of *R. irregularis* was inoculated to *Z. mays* in pot cultures with sterilized sand. After six months, the roots of *Z. mays* were collected and cut up with sterilized scissors. Then, the roots and sandy substrate were well mixed as inoculum.

The density of spores in the AM inoculum was measured following a previously published method [52]. Ten grams of inoculum were stirred in distilled water. After settling down the soil particles for 30 s, the supernatant was filtered by wet sieving (1 mm and 38 μm). The spores on the sieve were washed out by distilled water and extracted by density gradient centrifugation using a 50% sucrose solution. Then, the clean spores were placed on a white gridded cellulose nitrate filter (1.2 μm) and counted under a compound microscope (Olympus BX51, Tokyo, Japan).

### 2.2. Experimental Design

**Figure 1:**
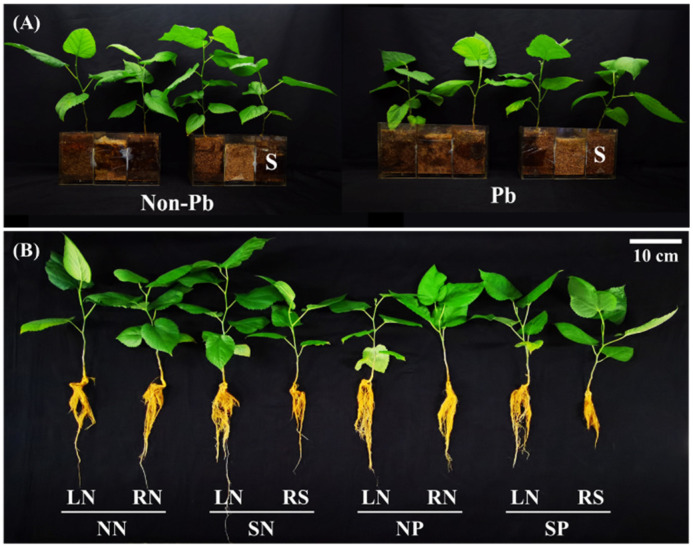
(**A**) The mulberries were shaded or unshaded in a three-compartment system and applied with/without Pb. S = shaded mulberry. (**B**) The mulberries were shaded or unshaded and applied with/without Pb. NN = unshaded treatment without Pb applied, control treatment; SN = shaded treatment without Pb applied; NP = unshaded treatment with Pb applied; SP = shaded treatment with Pb applied. LN = no Pb was added in the left unshaded mulberry; RN = the right unshaded mulberry without Pb added in the hyphal compartment; RS = the right shaded mulberry with Pb added in the hyphal compartment.

The experiment consisted of four treatments including no shading applied without Pb application in the hyphae compartment (NN), shading applied in one-side mulberry without Pb application in the hyphae compartment (SN), no shading applied with Pb application in the hyphae compartment (NP), and shading applied in one-side mulberry with Pb application in the hyphae compartment (SP). The mulberries in different compartments were labeled according to their positions (L: left; R: right) and shading status (as in Figure 1A,B). To assess the transfer capacity of Pb by the hyphae, 10 g AM inoculum was applied underneath the root of the mulberry for all treatments [27]. Each treatment contained three biological replications following a completely randomized design.

The shading application was four weeks after the AM inoculum application to ensure the AM fungal colonization and the formation of a hyphae bridge between the plants. The shading net was made of black plastic (40 cm × 40 cm × 60 cm) that blocked 80% of light (as shown in Supplementary Figure S2). Light intensity was recorded, ranging from 365 to 470 Lx for the shaded plants and 1840 to 2566 Lx for the unshaded plants. Three days after shading, 30 mL of 17.6 g·L^−1^ Pb (NO_3_)_2_ solution (Analytical reagent, Tianjin Damao, China) was added to the middle compartment (hyphal compartment) by syringe to reach 750 mg·kg^−1^ Pb in the substrate. The Pb (NO_3_)_2_ solution was added in the hyphal compartment at the same distance between the two outside compartments to avoid the dispersion of Pb solution.

Seedlings were grown in a greenhouse with 28 °C/24 °C day/night temperatures under 16 h daylight and 40–60% humidity. Thirty milliliters of modified Hoagland’s nutrient solution [53] containing 10% phosphate (0.1 mM KH_2_PO_4_) was added twice a week to each root compartment before Pb application. After Pb application, only water (30 mL) was added to the root compartment of all treatments every 2 days to avoid the direct precipitation of Pb.

### 2.3. Plant Sampling, Growth Status, and AM Fungal Colonization

At harvest (three weeks after Pb treatment), the height, ground diameter, biomass of shoots and roots, and fresh-to-dry mass ratio were measured. After measuring the fresh weights, parts of the shoots and roots were first dried in an oven at 105 °C with forced air circulation for 15 min to inactivate the enzymes, and then dried at 65 °C until they reached a constant weight for subsequent Pb measurement. Parts of the roots were fixed in FAA solution (37% formaldehyde:glacial acetic acid:95% ethanol, 9:0.5:0.5, *v*:*v*:*v*) for the assessment of the AM colonization [54]. Then, the root was cleared for 30 min in 10% KOH at 90 °C, acidified in 1% HCl for 3 min, and stained in 0.05% trypan following a previously published method [52]. The arbuscular and total colonization were measured using the magnified cross-section method [55]. The remaining parts of the shoots and roots were immediately frozen in liquid nitrogen and stored at −80 °C for the following analysis of gene relative expression.

### 2.4. Concentrations of Pb

The dry samples were ground in a mortar and then microwave digested with 65% nitric acid (MILESTONE DRN-41, Sorisole, BG, Italy) at 175 °C for 20 min according to the manufacturer’s instructions. The digestion solutions were then brought to 10 mL with 2% nitric acid. The Pb concentrations were measured using flame atomic absorption spectrometry (PerkinElmer PinAAciie 900F, Waltham, MA, USA) at 283.31 nm (analytical line). The Pb stock solution (1 g/L) of the standard was made according to GB/T 13080-2018 [56], and then diluted to 0.2, 0.4, 0.8, 1.6, 2, 4, 8, and 10 mg/L (the R^2^ value of the standard is 0.9996). The Pb content was calculated using the Pb concentration, fresh-to-dry mass ratio, and plant biomass to directly express the Pb extraction in the plant [57].

### 2.5. Photosynthesis

The third leaf of each plant was used for the photosynthetic measurements. On the harvest day from 8:00 to 10:30 a.m., the net photosynthetic rate (Pn), intercellular CO_2_ concentration (Ci), transpiration rate (Tr), and conduction to H_2_O (Gs) were measured using a Li-6400 portable open flow gas-exchange system (Li Corporation, Lincoln, NE, USA). The measurement conditions were summarized as follows: photosynthetically active irradiation, 1000 μmol·m^−2^·s^−1^; temperature, 27 °C; relative humidity, 30%; and CO_2_ concentration of sample cell, 350 μmol·mol^−1^.

### 2.6. Gene Relative Expression

The samples stored at −80 °C were ground and homogenized in liquid nitrogen with a mortar. Total RNA was extracted from samples by the E.Z.N.A.^TM^ Plant RNA Kit (Omega Biotech, Norcross, GA, USA) following the supplier’s instructions. After quantification of the RNA yield by a Nanodrop 2000 (Thermo Scientific, Pittsburgh, PA, USA), cDNA (complementary DNA) was synthesized from 1000 ng of RNA using a FastKing RT Kit with gDNase (TIANGEN Biotech, Beijing, China; gDNase, genomic DNase). The synthesized cDNA was diluted 5-fold and used as the template for PCR reactions.

The primers (the amplification efficiencies of the primers were within the range of 95% to 105%) used in the qRT-PCR are listed in Supplementary Table S1. The qRT-PCR reaction was conducted using the CFX96 real-time PCR detection system (Bio-Rad Laboratories, Hercules, CA, USA) and contained 5 μL ChamQ^TM^ Universal SYBR^®^ qPCR Master Mix (Vazyme Biotech, Nanjing, China), 0.5 μL (10 μM) forward primer, 0.5 μL (10 μM) reverse primer, 1 μL cDNA, and 3 μL ddH_2_O. The PCR procedure was as follows: an initial denaturation step at 95 °C for 3 min; 40 cycles of denaturation at 95 °C for 10 s, annealing at the specific temperature (specific annealing temperatures were in Supplementary Table S1) for 20 s, and extension at 72 °C for 20 s; a melting curve analysis of heating from 60 to 95 °C was carried out to check the specificity of the PCR amplification. The relative expressions of genes were normalized to the *M. alba* housekeeping gene *MaRPL* and *MaActin*, and *R. irregularis* housekeeping gene *RirEF1α*. The nucleotide sequences reported in this article have been submitted to GenBank and the accession numbers are listed in Supplementary Table S1. Duplicate samples were analyzed and negative controls without cDNA were run within each analysis. The relative quantity of transcripts was determined using the 2^−ΔCT^ method [58].

### 2.7. Statistical Analysis

The statistical analysis was performed using the SPSS 19.0 statistical program (SPSS Inc., Chicago, IL, USA). Analysis of variance (ANOVA) was used to test for significant differences in means across treatments (*F* and *p*-value of ANOVA are listed in Supplementary Table S3). When significant differences were found (*p* < 0.05), multiple comparisons were tested by Tukey’s test (N = 3) at a 0.05 significance level. Correlation analyses were analyzed by Spearman’s correlation (Supplementary Table S2). Different letters above the columns in the bar graph indicate a significant difference among the means by Tukey’s test (*p* < 0.05), while the same letters indicate an insignificant difference.

## 3. Results

### 3.1. Negative Effects of Pb and Shading on Plant Growth and Colonization

**Figure 2:**
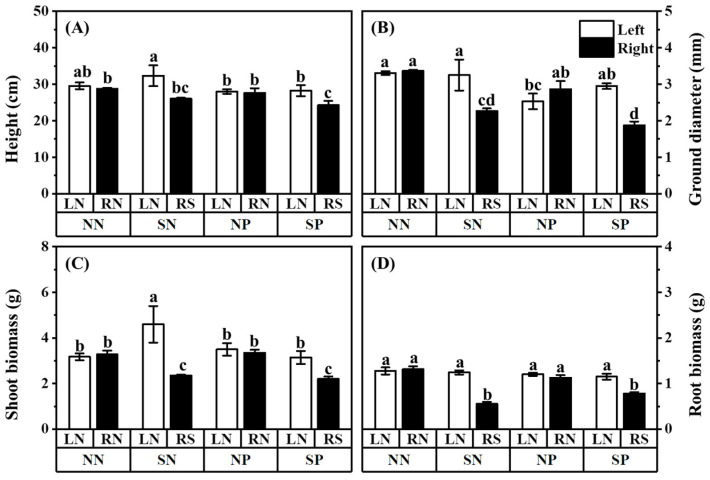
Effects of Pb and shading on the height (**A**), ground diameter (**B**), shoot biomass (**C**), and root biomass (**D**) in *M. alba*. The data are shown as the means ± standard deviation (*n* = 3). *F* and *p*-value of ANOVA are listed in Supplementary Table S3. Different letters above the columns in each graph indicate a significant difference among the means by Tukey’s test (*p* < 0.05). The abbreviation is consistent with Figure 1.

Three weeks after Pb application in the hyphal compartments, the biomass, height, and ground diameter of the mulberry were recorded (Figure 2). Shading reduced the height, ground diameter, shoot and root biomass of the shaded mulberry with Pb application (comparing treatment SP-RS with treatment SP-LN). The ground diameter, shoot and root biomass of the shaded mulberry without Pb application were also lower than that of the unshaded mulberry without Pb application. In the absence of Pb, the height and shoot biomass of the unshaded mulberry of the shade treatment were significantly higher than that in the control (Figure 2A,C; comparing treatment SN-LN with treatment NN-RN). There were no significant differences in the growth of shaded mulberries with and without Pb application (comparing treatment SN-RS with treatment SP-RS).

**Figure 3:**
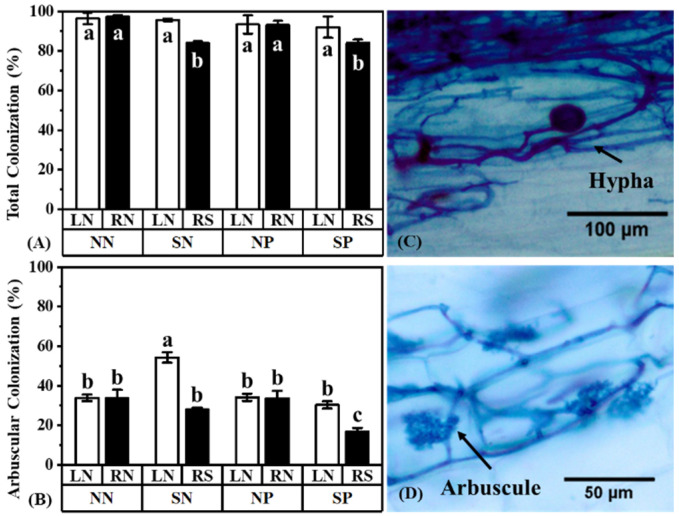
Effects of Pb and shading on the total colonization (**A**), arbuscular colonization (**B**), and the morphology of AM fungi (**C**,**D**) in *M. alba*. *F* and *p*-value of ANOVA are listed in Supplementary Table S3. Different letters above the columns in each graph indicate a significant difference among the means by Tukey’s test (*p* < 0.05). The abbreviation is consistent with Figure 1.

Over 90% of the root in the treatment NN (only inoculate with AM fungi) was colonized by AM fungi with the typical features of arbuscules (Figure 3). Shading significantly reduced the total colonization of the mulberry with or without Pb application and only reduced the arbuscular colonization of the mulberry with Pb application compared to the control (treatment NN-LN; Figure 3A). Meanwhile, shading increased the arbuscular colonization of the unshaded mulberry in the shade treatment without Pb application (Figure 3B; comparing treatment SN-LN with treatment NN-RN). The shaded mulberry with Pb application had lower arbuscular colonization than that observed in the shaded mulberry without Pb application (Figure 3B; comparing the treatment SP-RS with treatment SN-RS).

### 3.2. Negative Effects of Pb and Shading on Plant Photosynthesis

**Figure 4:**
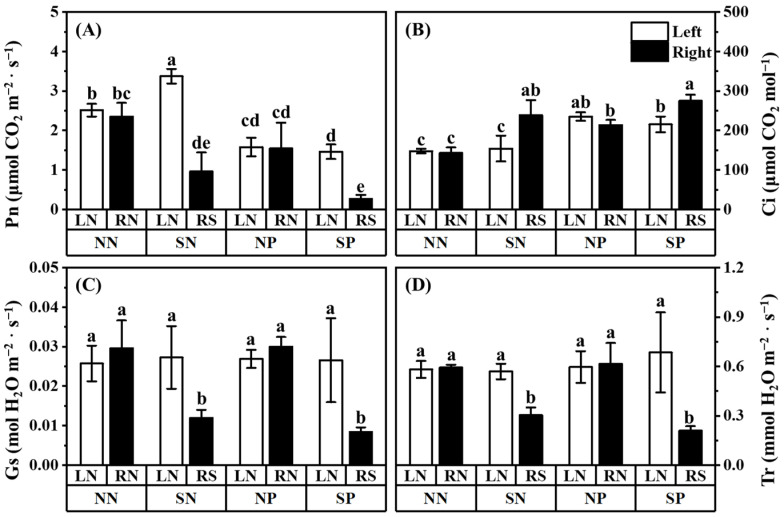
Effects of Pb and shading on the net photosynthetic rate Pn (**A**), intercellular CO_2_ concentration Ci (**B**), stomatal conductance Gs (**C**), and transpiration rate Tr (**D**) in the leaves of *M. alba*. *F* and *p*-value of ANOVA are listed in Supplementary Table S3. Different letters above the columns in each graph indicate a significant difference among the means by Tukey’s test (*p* < 0.05). The abbreviation is consistent with Figure 1.

Compared to the unshaded mulberry, regardless of whether or not Pb was applied, shading significantly decreased the Pn, Gs, and Tr of the shaded mulberry, but increased the Ci of the shaded mulberry (Figure 4). The application of Pb decreased the Pn but increased the Ci of both the shaded and unshaded mulberries (Figure 4A,B; comparing the treatment NN-LN with treatment NP-LN). Shading increased the Pn of the unshaded mulberry of shade treatment without Pb application (Figure 4A; comparing treatment SN-LN with treatment NN-RN). Among all the treatments, the lowest Pn, Tr, and Gs were observed in the shaded mulberry with Pb application (treatment SP-RS).

### 3.3. Pb Concentration and Content

**Figure 5:**
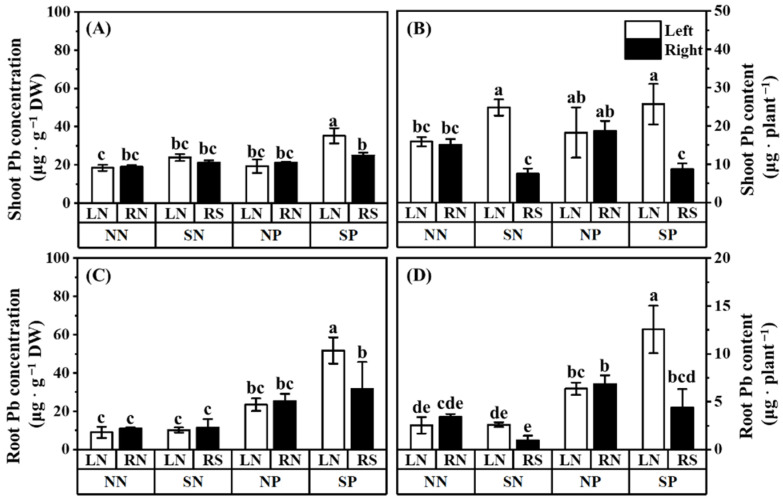
Effects of Pb and shading on the shoot Pb concentration (**A**), shoot Pb content (**B**), root Pb concentration (**C**), and root Pb content (**D**) in the leaves of *M. alba*. *F* and *p*-value of ANOVA are listed in Supplementary Table S3. Different letters above the columns in each graph indicate a significant difference among the means by Tukey’s test (*p* < 0.05). The abbreviation is consistent with Figure 1.

Shading significantly reduced the Pb content in the shoots and roots of the shaded mulberry (Figure 5B,D; comparing treatment SP-RS with treatment SP-LN). Interestingly, shading increased the shoot Pb concentration, root Pb content, and root Pb concentration of the unshaded mulberry of shade treatment with Pb application (Figure 5A,C,D; comparing treatment SP-LN with treatment NP-LN). The shoot Pb content of the unshaded mulberry of shade treatment without Pb application was also higher than the control (Figure 5B; comparing treatment SN-LN with treatment NN-LN). The application of Pb in the hyphal compartment increased the content and concentration of Pb in the root of mulberries in both the left and right compartments (Figure 5C,D). Among all of the treatments, the highest shoot and root Pb concentration and content were observed in the unshaded mulberry of shade treatment with Pb application (treatment SP-LN). There was no significant difference in the shoot Pb concentration and content of the shaded mulberry with or without Pb application.

### 3.4. Relative Expressions of Related Genes

**Figure 6:**
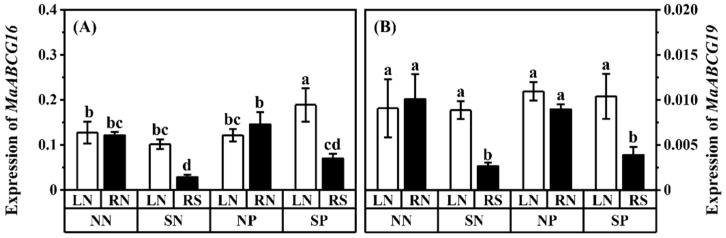
Expressions of *MaABCG16* (**A**)and *MaABCG19* (**B**) in roots of *M. alba*. *F* and *p*-value of ANOVA were listed in Supplementary Table S3. Different letters above the columns in each graph indicated a significant difference among the means by Tukey’s test (*p* < 0.05). The abbreviation is consistent with Figure 1.

ABCs often play important roles in transferring heavy metals [45,46,47]. The most numerous subtype transporter of the ABC transporter family is ABCGs, with the most complex function in a plant’s response to abiotic stresses [43,59]. Previous studies have shown that ABCGs play key roles in plant Pb tolerance [28,45,59,60], and *MaABCG16* and *MaABCG19* were upregulated by Pb and AM fungi (data unpublished). The application of Pb in the hyphal compartment slightly increased the expressions of *MaABCG16* and *MaABCG19* in the shaded mulberry compared to that in the unshaded mulberry (Figure 6; not significantly; comparing treatment SP-RS with treatment SN-RS). The expressions of *MaABCG16* and *MaABCG19* of the shaded mulberry were reduced by shading (Figure 6; comparing treatment SN-RS with treatment NN-RN, and treatment SP-RS with treatment NP-RN). Among all of the treatments, the highest expression of *MaABCG16* and *MaABCG19* was shown in the unshaded mulberry of shade treatments (Figure 6; treatment SP-LN).

**Figure 7:**
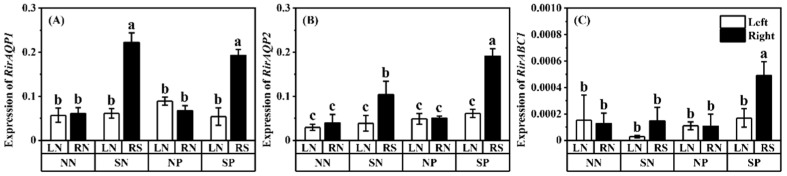
Expressions of *RirAQP1* (**A**), *RirAQP2* (**B**), and *RirABC1* (**C**) in roots of *M. alba*. *F* and *p*-value of ANOVA are listed in Supplementary Table S3. Different letters above the columns in each graph indicate a significant difference among the means by Tukey’s test (*p* < 0.05). The abbreviation is consistent with Figure 1.

To understand the effect of Pb and shading on the heavy metal tolerance and water transport of AM fungi, we measured the expressions of *RirABC1*, *RirAQP1*, and *RirAQP2*. Shading increased the expressions of *RirABC1*, *RirAQP1*, and *RirAQP2* of the shaded mulberry with or without Pb application (Figure 7; comparing treatment SN-RS with treatment NN-RN and treatment SP-RS with treatment NP-RN). The expressions of *RirAQP2* and *RirABC1* in shaded mulberry with Pb application (treatment SP-RS) were higher than that in shaded mulberry without Pb application (treatment SN-RS).

### 3.5. Correlation Analysis

According to the Spearman correlation analysis (Supplementary Table S2), shading significantly limited plant growth. The light intensity of the plant growth environment was added as an independent variable to explain the effect of shading. The light intensity showed positive correlations with the biomass, Pb content of the shoots and roots, height, ground diameter, Pn, Gs, Tr, expressions of *MaABCG16* and *MaABCG19*, and both the total and arbuscular colonization. In contrast, it showed negative correlations with Ci and the expression of *RirABC1*, *RirAQP1*, and *RirAQP2*.

The root Pb concentration showed negative correlations with the Pn but positive correlations with the root Pb content, shoot Pb concentration, and Ci. Moreover, the root Pb content showed positive correlations with the shoot Pb content, Tr, and the expression of *MaABCG16* and *MaABCG19*. The shoot Pb concentration showed positive correlations with the expression of *RirAQP2*. The shoot Pb content showed negative correlations with the expression of *RirAQP1* and *RirAQP2*, but positive correlations with the shoot biomass, height, ground diameter, Pn, Gs, Tr, the expression of *MaABCG16* and *MaABCG19*, and both the total and arbuscular colonization.

## 4. Discussion

Arbuscular mycorrhizal fungi have been proven to improve the growth and Pb tolerance of host plants [27,61]. The AM fungi colonized plants were observed to accumulate more Pb in the roots than in the shoots [28,62], and especially accumulate Pb in the mycorrhizal colonized root segment [27]. To verify the contribution of AM extraradical hyphae on the Pb uptake of host plants with different light intensities, a network of AM extraradical hyphae was established in this study to link two plants with the same or different light intensities.

### 4.1. The AM Fungi Impact More on High-Light Mulberry than Low-Light Mulberry

The similar biomass, height, and ground diameter between two unshaded mulberries without Pb application in the three-compartment systems indicated that the hyphae transported nutrients evenly to the mulberries on both sides. It also demonstrated the success of building a hyphae network to link two mulberries using the three-compartment systems. Shading decreased the light intensity (Supplementary Figure S2B) and inhibited the photosynthesis of mulberry (Figure 4A), which reduced the mulberry ground diameter and the biomass of the shoots and roots (Figure 2C,D), consistent with the observations in previous studies [63,64].

Interestingly, the unshaded mulberry in the shaded treatment (SN-LN) had better growth than the unshaded mulberry in the unshaded treatment (Figure 2A,C; treatment NN-LN), which indicated that *R. irregularis* might prefer to transfer nutrients to high-light mulberry rather than low-light mulberry. Light is a critical factor that can substantially affect the transfer of photosynthetic products—C compounds—from the shoots to the roots in mycorrhizal plants [63]. Our results demonstrated the importance of plant light status in the nutrient exchange between AM fungi and plants [20] where *R. irregularis* might prefer to allocate more nutrients to unshaded mulberry with high C payback rather than to shaded mulberry with low C payback. However, this phenomenon was not observed when Pb was applied in this study. The shoot biomass and height of the unshaded mulberry of the shaded treatment with Pb application (treatment SP-LN) was not significantly higher than that of the unshaded treatment with Pb application (Figure 2A,C; treatment NP-LN). This indicated that exposure to Pb inhibits the positive effect of *R. irregularis* on the growth of mulberry and might inhibit the nutrient transport of *R. irregularis* extraradical hyphae.

Previous studies have proven that plants can acquire N, P, and K through the symbiotic uptake pathway [65,66,67]. Among the major common nutrients, the bioavailability of P is usually one of the main factors limiting plant growth [68,69,70]. The P delivered through the extraradical hyphae of *R. irregularis* might be inhibited by Pb due to the formation of a stable, insoluble Pb (PO_4_)_2_ with P [71]. Based on results from previous studies, we assumed that although *R. irregularis* might deliver more P to unshaded plants than to shaded ones, this delivery could be inhibited by the presence of Pb as a high Pb concentration was observed in the roots of unshaded mulberry of shade treatment with Pb application (Figure 5C; treatment SP-LN). Therefore, the inhibition of P transfer by the presence of Pb might be the main reason for the inhibition of nutrient transport by *R. irregularis* to high-light state mulberry with Pb application. Further research is needed to clarify this.

### 4.2. The Status of Plant Light and Pb Effect AM Fungal Colonization

Under heavy metal stressed environments, AM fungi often protect the host plants from toxicity by improving the heavy metal tolerance of host plants [26,27] and modulating the plant antioxidant enzyme [28,29]. In contrast, the presence of heavy metals in the growth substrates can reduce AM fungal hyphae extension in vitro [72]. Exposure to Pb often decreased the colonization of AM fungi [31,73]. However, in this study, the root was not exposed to Pb during the colonization and formation of the AM fungal hyphae network, together with the results that no correlation between the Pb concentrations of mulberry and the total colonization of *R. irregularis* was observed (Supplementary Table S2), demonstrating that Pb application did not affect the colonization of *R. irregularis* when symbiosis had already formed (Figure 3). The total and arbuscular colonization showed a similar trend between the non-Pb treatment NN and Pb treatment NP (Figure 3A,B), indicating that the colonization of *R. irregularis* was not inhibited when the colonization environment had no Pb (the Pb was applied in the hyphal compartment in the later stage of the experiment).

After shading, C sources available from mulberry to *R. irregularis* were inhibited, and the total and arbuscular colonization greatly decreased (Figure 3). Shading reduced the photosynthetic capacity and C allocation to roots, which might inhibit the efficiency of C and nutrient exchange between plants and AM fungi [63,74]. Several studies have also shown that plant investment in mycorrhizal fungi decreased as light decreased [48,49]. Plants reduced their C investment in fungi as the C they gained through photosynthesis decreased. The decrease in the total and arbuscular colonization after shading reflected the plant’s strategy of reducing the C investment in fungi. To deal with the reduced C investment from the host plant, AM fungi prefer to form a symbiosis with plants that can provide plenty of C sources in the same AM hyphal network. This also showed that the status of mulberry, especially the C supply, had a great impact on the colonization of *R. irregularis*.

### 4.3. The AM Fungi Prefer to Transfer Pb to High-Light Mulberry Rather than Low-Light Mulberry

Arbuscular mycorrhizal fungi can stimulate the accumulation of Pb in their host plants [26,28,75,76]. The Pb concentration and content in the root of unshaded mulberries in shade treatment (treatment SP-LN) were higher than that in the control (treatment NP-LN) when Pb was applied in the hyphal compartment (Figure 5C,D), suggesting that *R. irregularis* in the hyphal compartment prefer to transfer Pb to the high-light mulberry rather than low-light mulberry. Considering that the light status directly affects the photosynthetic efficiency of mycorrhizal plants and consequently affects the transfer of C compounds from the shoots to roots in mycorrhizal plants [63] and the element exchange between AM fungi and plants [20], the photosynthetic efficiency of the host plant should be responsible for the preference of *R. irregularis* on Pb transfer. The *R. irregularis* might prefer allocating more Pb to high-light plants (i.e., unshaded mulberry in shade treatment) with high C payback rather than to low-light plants (i.e., shaded mulberry) with low C payback. This is supported by the unaltered shoot Pb concentration of the shaded mulberry in the Pb treatment (compare treatment SP-RS with SN-RS, Figure 5A). When the photosynthetic efficiency was low, the Pb in the root hardly moved. For most treatments, this portion of Pb was not transferred to the shoot (Figure 5A).

However, there was a significant increase in the shoot Pb concentration in the treatment SP-LN (only the unshaded mulberry of shaded treatment with Pb application; Figure 5A). The increase also indicated that there might be a threshold for Pb fixation by AM intraradical hyphae (Figure 5A). Below a threshold concentration, the Pb in the roots of AM mulberry might be fixed on the intraradical hyphae. However, beyond this threshold value, the absorbed Pb in the AM fungal intraradical hyphae would transfer to the mulberry root and even to the shoot. This was further supported by the higher root Pb concentration in treatment SP-LN compared to treatment SP-RS (the shaded mulberry under Pb application). We also observed that the root Pb content in treatment SP-LN was significantly higher than that in the control. This increment might be due to increased biomass and photosynthetic efficiency (Figure 2 and Figure 4A). Correlation analysis also showed that the Pb content of the mulberry shoot was significantly positively correlated with light intensity, Pn, Tr, and shoot biomass (Supplementary Table S2). Our results showed that mulberry with high photosynthetic efficiency could grow better and absorbed more Pb than that with low photosynthetic efficiency. Considering the fact that AM plants often had higher photosynthetic efficiency compared to non-AM plants [23,24,25], our results imply that the amount of Pb extracted from the soil by AM plants is associated with their photosynthetic efficiency. This also provides new insights into the application of mycorrhizal mulberry with high photosynthetic efficiency in the remediation of heavy metal polluted environments.

### 4.4. Effect of Shading and Pb on Related Gene

It has been reported that ABCGs play key roles in plant Pb tolerance [28,59,77]). Overexpression ABCGs of *A. thaliana* resulted in increased Pb tolerance [45]. From our previous study, *MaABCG16* and *MaABCG19* were specifically upregulated by *R. irregularis* (data not published) and played key roles in Pb tolerance. In this study, the expression of *MaABCG16* was higher in the unshaded mulberry of shaded treatment with Pb application (treatment SP-LN) than in the control with a similar trend in thee shoot Pb concentration, suggesting that the gene might play an important role in Pb translocation from root to shoot (Figure 6). To explore the response of AM fungi to Pb and shading, the ABC genes of AM fungi were also investigated. The expression of ABC genes of AM fungi was found to be upregulated by Cd and Cu in a previous study, suggesting that this transporter might be involved in heavy metal detoxification [47]. The expression of *RirABC1* in shaded mulberry root with Pb applied was upregulated, indicating the importance of this gene for Pb translocation (Figure 7C). According to Figure 6, shading suppressed ABC protein expression while it inhibited Pb transfer. This might be because shading inhibited plant photosynthesis and ATP synthesis [78,79,80]. The compound exchange driven by ABC proteins requires the use of energy released by ATP hydrolysis [81]. Thus, the expression of *MaABCG16* and *MaABCG19* might be inhibited by shading, as observed in this study. Our study indicates that the response of *R. irregularis* to mulberry Pb uptake was closely related to the ABCs and might be affected by the difference in mulberry photosynthesis.

It is known that AM fungi are capable of transferring Pb to the roots of host plants by stimulating transpiration flow [27], but the relationship between water transport and Pb transport of AM fungi is still unclear. To explore the response of AM fungi to Pb and shading, the AQP genes of AM fungi were investigated. The AM fungal aquaporins have been related to water transport in the extraradical hyphae and across the peri arbuscular membrane [82] and play key roles in nutrient translocations [42]. Shading promoted the expression of *RirAQP1* and *RirAQP2* with or without Pb application, suggesting that the water transport of *R. irregularis* was stimulated after the plants were shaded (Figure 7A,B). Hu et al. [83] also found that *RirAQP1* and *RirAQP2* were upregulated by water stress. This might be the strategy of *R. irregularis* responding to abiotic stress (i.e., light and water stress). *R. irregularis* might need to obtain nutrients from other sources when insufficient light in mulberry results in insufficient C supply to *R. irregularis*, or need to seek water when under water stress conditions. The AM fungal aquaporins were stimulated to transport more water and obtain more nutrients from surrounding environments other than from the host plants. A similar compensation mechanism by the AM fungi was revealed by Van’t Padje et al. [84]. The expressions of these genes (*RirAQP1*, *RirAQP2*, and *RirABC1*) of *R. irregularis* were upregulated by shading and were significantly negatively correlated with light intensity (Supplementary Table S2). These observations suggest that underground fungal activities may be facilitated rather than suppressed by shading, which might be directly related to the balance of material exchange between *R. irregularis* and mulberry. Further systematic study is warranted to explore how these fungal genes are tuned to respond to stress when plant C supply is reduced.

## 5. Conclusions

This study demonstrated that *R. irregularis* tended to transfer more Pb to high-light mulberry, a condition capable of providing more C supply for fungal survival, than to low-light mulberry. Nutrient delivery by *R. irregularis* to high-light mulberry might be inhibited in the presence of Pb. When the C supply of host mulberry is insufficient, *R. irregularis* would seek other nutrient sources from environments other than from the host mulberry by regulating the expression of *RirAQP1* and *RirAQP2*. Furthermore, the Pb transferred through the mycorrhizal pathway had low mobility and was likely to remain in *R. irregularis* of mulberry root until a threshold was exceeded. Beyond this threshold value, the absorbed Pb, mainly in AM fungal intraradical hyphae, might be transferred to the plant root and even to the shoot. The high expression of *MaABCG16* with high root and shoot Pb concentration suggests that *MaABCG16* plays a critical role in Pb translocation. The specific role of *MaABCG16* in fungal and plant Pb transfer deserves further study.

## Data Availability

Not applicable.

## Supplementary Materials

The following supporting information can be downloaded at: https://www.mdpi.com/article/10.3390/jof8111224/s1, Figure S1: The details of the three-compartment system used in this study. Figure S2: The shading device used in this experiment (**A**) and the light intensity of the shaded and unshaded environment (**B**). The top light intensity was measured at the top of the mulberry. The bottom light intensity was measured at the base of the mulberry without leaf sheltering. Table S1: Primer list of RT-qPCR. Table S2: Correlation analysis between AM fungi colonization in the root, Pb, and light intensity with biomass, photosynthesis, and the relative expressions of related genes in different treatments. Table S3: ANOVA analysis.
