# Supplementary figures and images for: Pb Transfer Preference of Arbuscular Mycorrhizal Fungus Rhizophagus irregularis in Morus alba under Different Light Intensities
Source: J Fungi (Basel). 2022 Nov 20;8(11):1224. doi: 10.3390/jof8111224 (PMC9694238; doi:10.3390/jof8111224)

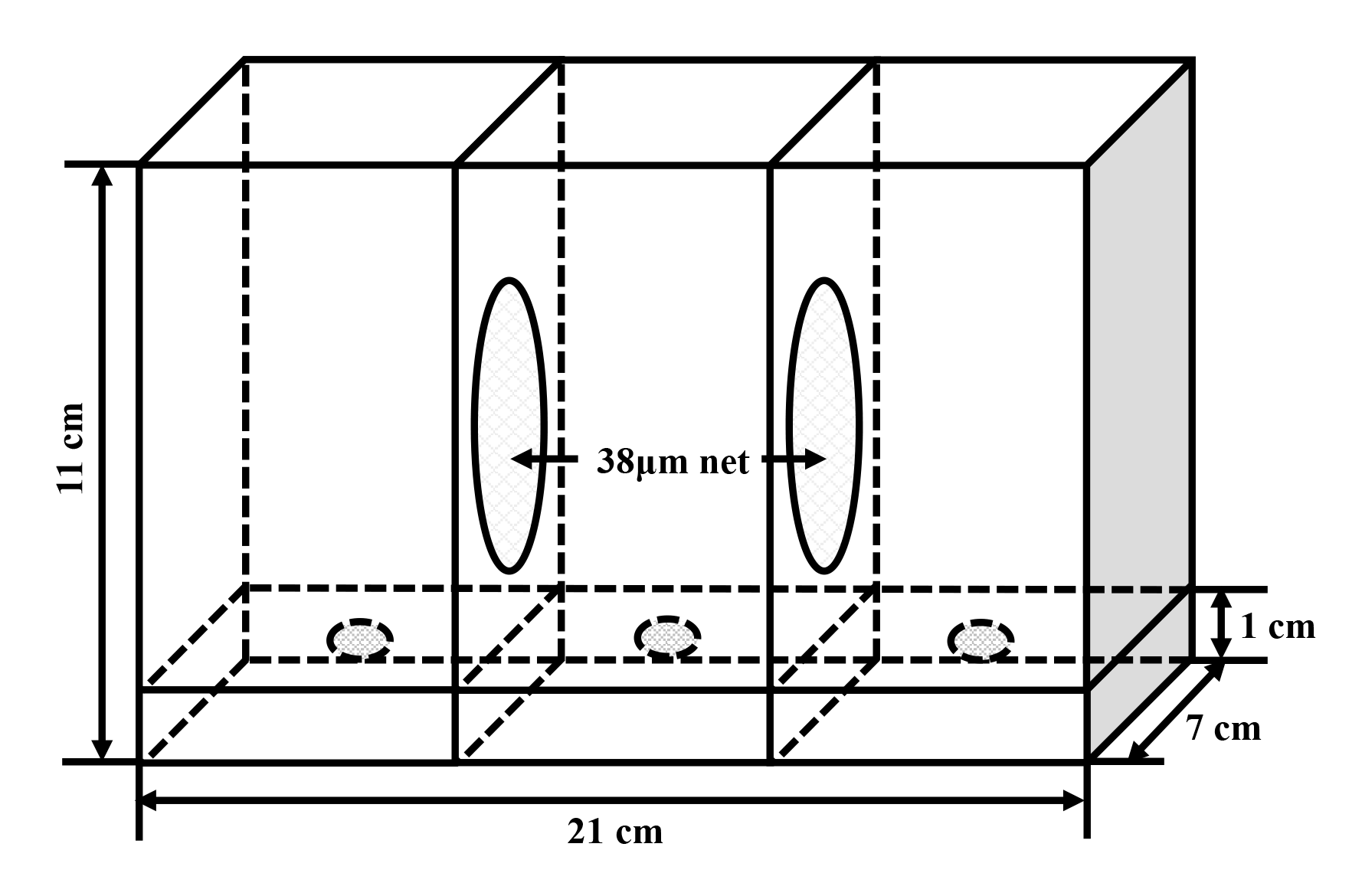

Supplement: Supplementary file 1 [file jof-08-01224-s001.zip › Supplementary/Supplementary Figure S1.tif]

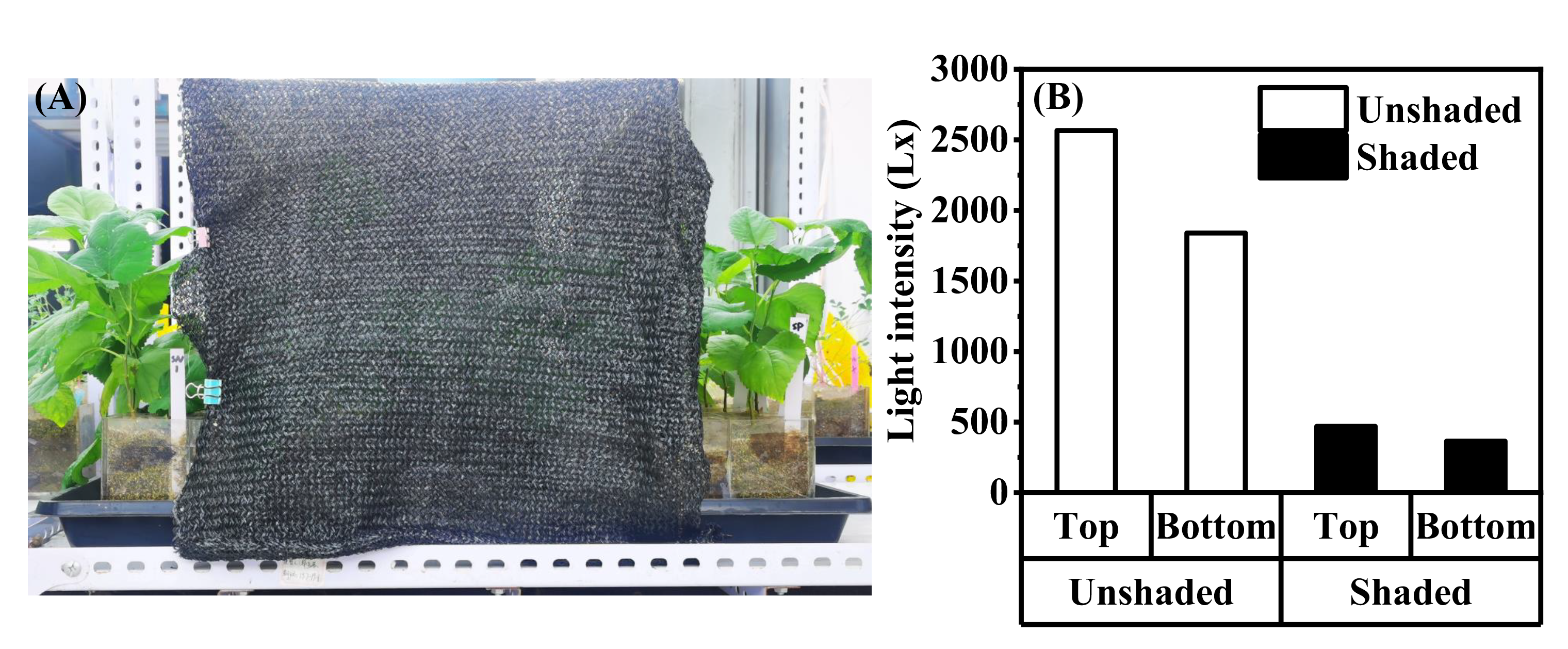

Supplement: Supplementary file 1 [file jof-08-01224-s001.zip › Supplementary/Supplementary Figure S2.tif]
